# Targeting Necroptosis: A Novel Therapeutic Option for Retinal Degenerative Diseases

**DOI:** 10.7150/ijbs.77994

**Published:** 2023-01-01

**Authors:** Qi Zhang, Xi-min Hu, Wen-juan Zhao, Xiao-xia Ban, Yan Li, Yan-xia Huang, Hao Wan, Ye He, Lv-shuang Liao, Lei Shang, Bin Jiang, Guo-ping Qing, Kun Xiong

**Affiliations:** 1 Department of Anatomy and Neurobiology, School of Basic Medical Sciences, Central South University, Changsha, China.; 2 Key Laboratory of Emergency and Trauma, Ministry of Education, College of Emergency and Trauma, Hainan Medical University, Haikou, China.; 3 Changsha Aier Eye Hospital, Changsha, China.; 4 School of Physical Education, Hunan Institute of Science and Technology, Yueyang, China.; 5 Affiliated Eye Hospital of Nanchang University, Jiangxi Research Institute of Ophthalmology and Visual Science, Jiangxi Clinical Research Center for Ophthalmic Disease, Nanchang, China.; 6 Department of Ophthalmology, The Second Xiangya Hospital, Central South University, Changsha, China.; 7 Beijing Tongren Eye Center, Beijing Tongren Hospital, Capital Medical University, Beijing, China.; 8 Beijing Ophthalmology and Visual Sciences Key Laboratory, Beijing, China.; 9 Hunan Key Laboratory of Ophthalmology, Changsha, China.

**Keywords:** Retinal degenerative diseases, necroptosis, RIPK1, RIPK3, age-related macular degeneration, glaucoma

## Abstract

The discovery of the necroptosis, a form of regulated necrosis that is mediated by receptor-interacting protein kinase 1 (RIPK1), RIPK3, and mixed-lineage kinase domain-like pseudokinase (MLKL), represents a major breakthrough that has dramatically altered the conception of necrosis - traditionally thought of as uncontrolled cell death - in various human diseases. Retinal cell death is a leading cause of blindness and has been identified in most retinal diseases, e.g., age-related macular degeneration, glaucoma, retinal detachment, retinitis pigmentosa, *etc*. Increasing evidence demonstrates that retinal degenerative diseases also share a common mechanism in necroptosis. Exacerbated necroptotic cell death hinders the treatment for retinal degenerative diseases. In this review, we highlight recent advances in identifying retinal necroptosis, summarize the underlying mechanisms of necroptosis in retinal degenerative diseases, and discuss potential anti-necroptosis strategies, such as selective inhibitors and chemical agents, for treating retinal degenerative diseases.

## Introduction

Retinal cell death is a leading cause of vision impairment and blindness in a variety of retinal diseases 1-3. Severe microenvironmental changes caused by retinal disease and injury can lead to the degeneration of retinal cells 4-6. These microenvironmental changes include, but are not limited to, oxidative stress, hypoxic/ischemic stress, and excessive glutamate 7-9. Mutations in genes encoding enzymes and retinoid-binding proteins also result in retinal cell death in inherited retinal diseases 10, 11.

According to different responses to various pathological stresses/genetic defects, cells may die from accidental cell death (ACD, also called necrosis) or from regulated cell death (RCD), a well-controlled process involving a variety of signaling cascades or effectors 12, 13. Degeneration of retinal cells has been thought to occur through apoptosis, which is the most studied type of RCD mediated by activation of caspases (caspase-3/7/8/9) 14. Apoptosis is characterized by cell shrinkage, nuclear condensation, and membrane blebbing without release of cellular contents 15. Thus, apoptosis usually does not elicit an inflammatory response and pathological subsequences 16.

Autophagy is a self-degradative process that contributes to removing damaged cellular components, proteins, and inflammatory cytokines 17. Excessive rates of autophagy can result in an autophagy-dependent cell death through excessive catabolism 18. However, the role of autophagy in retinal degenerative diseases is debated. Autophagy in glaucoma promotes autophagic cell death of retinal ganglion cells (RGCs) 19. In contrast, impairment of autophagy has been reported in patients with age-related macular degeneration (AMD), which causes the retinal pigment epithelium (RPE) cells to fail to sufficiently resist oxidative damage, thereby leading to RPE degeneration 20.

Unlike apoptosis and autophagy, pyroptosis is a pro-inflammatory form of RCD, characterized by cell swelling, membrane rupture, and inflammatory cytokine release 21. Pyroptosis is initiated by activation of the NOD-like receptor family pyrin domain-containing 3 (NLRP3) inflammasome complex 22. Thereafter, activated caspase-1 from the complex cleaves gasdermin D (GSDMD) to induce the formation of cell membrane pores that facilitate cell rupture and release of intracellular cytokines 23. Although pyroptosis and apoptosis are both regulated by caspases, caspase inhibition is not sufficient to hinder or prevent retinal cell loss, indicating that other forms of cell death are also involved 24.

Necroptosis is a form of regulated necrotic cell death governed by receptor-interacting protein (RIP) kinases (RIPKs), which contribute to the pathogenesis of neurodegenerative diseases 25, 26. Emerging evidences indicate that necroptosis is a common mechanism of retinal cell death in addition to apoptosis 14, 27, 28. Although the clinical manifestation and pathogenesis of each retinal disease differ, the necroptotic signaling pathways seem to be shared among them, at least in part, and inhibition of necroptosis provides efficient neuroprotective effects in the treatment of multiple retinal degenerative disorders 29-31.

In the present review, we summarize the current understanding of necroptotic mechanisms and their roles in retinal diseases and injuries. We also highlight key molecules involved the necroptosis of retinal cells and the respective inhibitors that promise to produce new therapeutic strategies for the management of retinal degenerative diseases.

## Molecular mechanisms of necroptosis

### RIPK1 dominates which to happen: apoptosis or necroptosis

**Figure 1:**
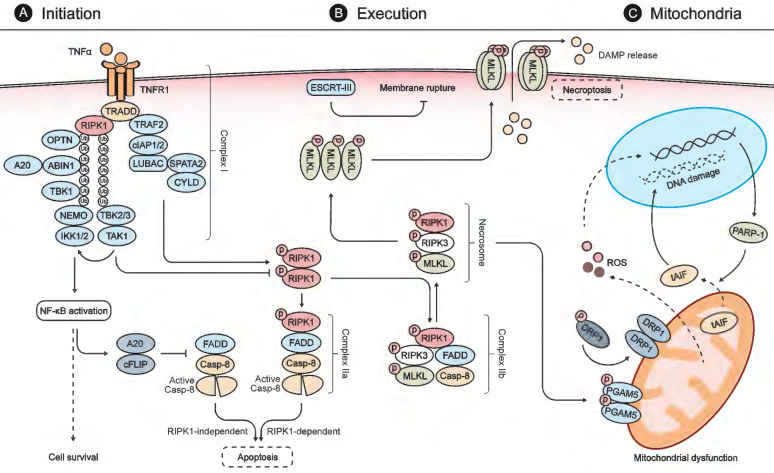
** Molecular mechanisms of necroptosis. (A)** Stimulation of TNFR1 by TNFα promotes the formation of a membrane-associated protein complex (complex I), which is composed of TRADD, RIPK1, TRAF2, cIAP1/2, LUBAC, TAK1, and the IKK complex. In complex I, cIAP1/2 and LUBAC promote RIPK1 ubiquitination that recruits TAK1, TAB2/3, and the IKK complex composed of IKK1/2 and NEMO. Complex I can induce expressions of both pro-survival and pro-inflammatory genes *via* NFκB activation. NFκB activation, in turn, blocks the formation of FADD and caspase-8 complex (also called complex IIa-RIA) through A20 and cFLIP to modulate RIPK1-independent apoptosis. When complex I is unstable or the ubiquitination of RIPK1 is inhibited, stimulation of TNFR1 leads to the dimerization and activation of RIPK1, which interacts with FADD and caspase-8 to form complex IIa-RDA and mediates subsequent apoptosis. When the activity of caspase 8 is blocked, the activated RIPK1 recruits RIPK3 and MLKL, leading to the formation of complex IIb and subsequent phosphorylation of MLKL. **(B)** In the necrosome, RIPK3 phosphorylates MLKL and leads to its activation and oligomerization. Then the oligomerized MLKLs translocate to the plasma membrane to form a pore-forming complex, causing plasma membrane rupture and release of DAMP molecules, which provoke further inflammation and secondary injury. ESCRT-III, a downstream regulator of MLKL, can counteract a limited amount of plasma membrane perforation mediated by MLKL through shedding of the affected segments. **(C)** During the process of necroptosis, the necrosome can also phosphorylate PGAM5 and lead to a PGAM5-mediated mitochondrial dysfunction. The activated PGAM5 dephosphorylates DRP1 and causes its translocation from the cytosol to the mitochondrion to stimulate mitochondrial fission and ROS generation. The increasing levels of ROS subsequently induce PARP1 activation, which stimulates the release of tAIF from the mitochondria to the cytosol and, from there, to the nucleus, which provokes DNA degradation and subsequent cell damage.

Necroptosis is initiated by diverse stimuli detected by specific death receptors which include, but are not limited to, tumor necrosis factor (TNF)-receptor 1 (TNFR1), FAS receptor, and Toll-like receptors (TLRs) 12, 25. These death receptors commonly include a death domain (DD) that can recruit RIPK1 by homotypic binding following stimulation by their respective cognate ligands (Fig. 1) 25. The TNF-mediated signaling pathway is one of the most studied pathways for necroptosis. Following binding of TNF-α to TNFR1, TNFR1-associated DD protein (TRADD) recruits RIPK1 to initiate the formation of a membrane-associated protein complex (complex I) to induce direct pro-inflammatory signaling *via* the activation of nuclear factor-κB (NF-κB). This inflammatory signaling cascade blocks the formation of cytosolic complex IIa, comprised of FADD and caspase-8, which would otherwise modulate RIPK1-independent apoptosis 25, 32, 33. If RIPK1 is allowed to dimerize by blocking its ubiquitination, the activated RIPK1 dimer can interact with FADD and caspase-8 to form complex IIa, which mediates apoptosis 25, 33. Under certain conditions, when cells are defective in activating caspase-8 or when RIPK3 is overexpressed, the activated RIPK1 recruits RIPK3 and MLKL, leading to the formation of complex IIb and subsequent execution of necroptosis 25, 33, 34.

### Oligomerization and translocation of MLKL executes necroptosis

Phosphorylation of MLKL by the RIPK3 kinase domain is required for MLKL to function in the execution of necroptosis 35-37. RIPK3 recruits and phosphorylates MLKL, leading to the latter's oligomerization and subsequent translocation to the plasma membrane, where it assembles into a pore-forming complex and causes loss of plasma membrane integrity 32, 36, 37. The cell rupture caused by necroptosis can promote inflammation and secondary injury by releasing intracellular damage-associated molecular patterns (DAMPs) such as high-mobility group box 1 (HMGB1) protein 38. This process can be counteracted by the endosomal sorting complex ESCRT-III, which can facilitate the shedding of sections of MLKL-embedded plasma membrane from intact cells 37, 39.

### Mitochondrial mechanisms involving necroptosis

The mitochondria are the intracellular sites where ATP is generated to meet sustained cellular energy demands 40. Mitochondria are also critical for regulation of apoptosis and necrosis *via* different mechanisms 41. Induction of mitochondrial outer membrane permeabilization (MOMP) by BAX/BAK leads to the release of cytochrome c to promote caspase activation and apoptosis 42. In contrast, opening of the mitochondrial permeability transition pore (mPTP) in the inner mitochondrial membrane (IMM) is a major event of necrosis that shuts down ATP production and leads to cellular energy starvation 18.

Mitochondrial dysfunction is also involved in necroptosis 40. The RIPK1/RIPK3 necrosome can activate phosphoglycerate mutase family member 5 (PGAM5) 43. PGAM5 can, in turn, cause the translocation of dynamin-related protein 1 (DRP1) from the cytosol to the mitochondrion to stimulate mitochondrial fission and reactive oxygen species (ROS) generation, both of which are early steps for the execution of the necroptosis 38, 43. The mitochondrial protein apoptosis-inducing factor (AIF), a protein vital to respiratory chain stability and/or to maintenance of the mitochondrial structure, is also involved in the mitochondrial mechanisms of necroptosis 44. Poly (ADP-ribose) polymerase 1 (PARP1) activation stimulates the release of truncated AIF from the mitochondria to the cytosol 44, 45. Truncated AIF then rapidly translocates to the nucleus to provoke DNA degradation and subsequent cell damage. Notably, it was reported that the translocation of AIF could be attenuated by inhibition of RIPK1 during acetaminophen-evoked necroptosis, suggesting a crosstalk between TNFR1- and mitochondria-mediated necroptosis 46. These key molecules mentioned above have been widely applied in characterizing necroptotic cell death in retinal degenerative diseases and discovering potential targeted therapeutic strategies.

## Methods to detect necroptosis in retinal cells

### Cell viability assays

The assessment of cell viability is fundamental to *in vitro* cell stress research 47, 48. In combination with the treatment of necroptosis inhibitors, cell viability assays are important tools to estimate necroptosis-related injury to retinal cells in *in vitro* models of retinal degenerative diseases. Such assays include the 3-(4,5-dimethylthiazol-2-yl)-2,5-diphenyl tetrazolium bromide (MTT) assay and 4',6-diamidino-2-phenylindole (DAPI)/Hoechst staining 28, 49-51. The *in vitro* cell viability of RGCs can also be measured by the release of lactate dehydrogenase, which rapidly increases in cell culture medium upon plasma membrane rupture following retinal ischemia-reperfusion (I/R) injury 28.

### Morphological changes

**Figure 2:**
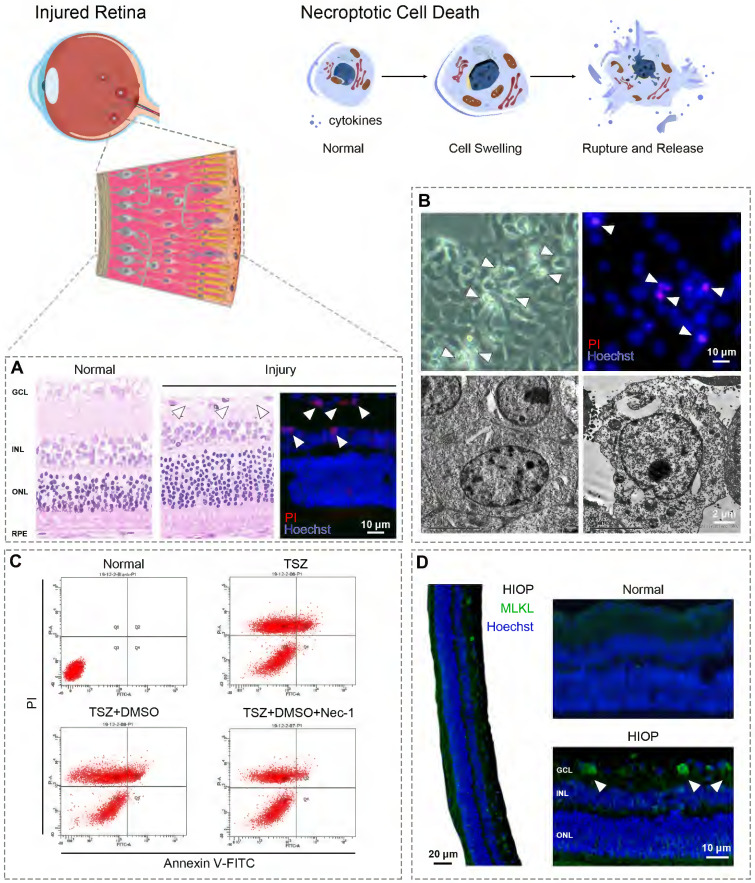
** Detecting the necroptosis in retina. (A)** Cell loss occurs widely in different retina injuries/diseases and leads to visual impairment, owing to ischemia/reperfusion, excitotoxicity, and inflammatory reactions. In rat retinas following high intraocular pressure (HIOP) injury (an *in vivo* animal model of retinal ischemia/reperfusion), hematoxylin and eosin staining indicated significant cell loss in the ganglion cell layer caused by excessive pressure. Cell membrane rupture played a critical role in the cell loss caused by HIOP injury - a notable observation, given that membrane rupture is a typical feature of necroptosis. **(B)** *In vitro* observation of R28 cells (a retinal precursor cell line) provides a feasible way to show retinal cell swelling (the other hallmark of necroptosis; upper left) and membrane rupture (upper right) following oxygen-glucose deprivation/recovery injury (an *in vitro* cell model of retinal ischemia/reperfusion). TEM enables an ultra-high-resolution characterization of retinal cell swelling and membrane rupture of necroptotic cell death (lower right) compared with the normal microscopy (lower left). **(C)** TNFα/Smac-mimetic/Z-VAD-FMK (TSZ)-induced cell necroptosis is a classical tool to investigate necroptotic signaling. As indicated by flow cytometry with propidium iodide/Annexin V staining, TSZ and TSZ+DMSO treatment showed a large number of necrotic cells (PI positive; upper left and/or right quadrants of each panel) compared with the normal group. Necrostatin-1 (Nec-1), a selective inhibitor of RIPK1 that can reverse necroptotic cell death, is widely used as a positive control to determine the existence of necroptosis by flow cytometry. **(D)** Upregulation of RIPK1, RIPK3, and MLKL indicated by immunofluorescence or immunohistochemistry are key biomarkers to identify necroptotic cell death. As showed by immunofluorescence assay, the expression of MLKL was significantly enhanced in retinal ganglion cells following HIOP injury, indicating the presence and execution of necroptosis.

Necroptosis shows similar morphological features to necrosis. Both are characterized by cytoplasmic swelling, plasma membrane rupture, and release of intracellular contents (Fig. 2) 12. In contrast, apoptosis is marked by plasma membrane blebbing and apoptotic body formation, while autophagy-dependent cell death is characterized by accumulation of double membrane vesicles called autophagosomes 15, 18. Although pyroptosis exhibits similar morphological changes to necroptosis, the two can be distinguished by specific inhibitors of their respective master regulators 38, 52. Transmission electron microscopy (TEM) is usually used to identify the necroptotic morphology of retinal cells 14, 53-55. For example, in retinal tissues from glaucoma patients, ultrastructural changes in RGCs revealed by TEM showed typical necroptotic morphology such as cell swelling and membrane rupture 56.

Fluorescence microscopy is also used to identify the plasma membrane rupture typical of necroptosis through propidium iodide (PI) staining *in vitro* and* in vivo* 28, 57, 58. It's important to note that distinguishing necroptosis from necrosis and other PCD *via* PI and/or Annexin V staining must be paired with specific necroptotic inhibitors 28, 57.

### Genetic features

Upregulated phosphorylation of RIPK1/3 and MLKL can also be used as an indicator of necroptosis 59. In retinal tissue from glaucoma patients, the upregulated expression of RIPK1 in retinal cells was used to assess necroptosis indicated by immunohistochemistry 56. Notably, the elevated MLKL immunofluorescence was applied as a typical necroptotic marker in AMD human retina sections 60. The upregulated phosphorylation of RIPK1/3, and MLKL was also considered as key indicator of necroptosis in retinal cells 14, 28, 29. Of note, RIPK1 plays multiple roles in necroptosis, apoptosis and inflammatory pathways; thus, upregulation of RIPK1 is insufficient to define necroptosis. Rather, detection must be made in combination with RIPK3 and/or MLKL expression, preferably along with inhibitors of other types of RCD 34, 61.

## Necroptosis drives death of retinal cells in retinal diseases and injuries

### Age-related macular degeneration

Age-related macular degeneration (AMD) is a major cause of irreversible visual loss in elderly people worldwide 20, 62. The etiology of AMD is complicated, consisting of lipofuscin accumulation, chronic inflammation, and dysfunctional ocular microcirculation, to name just some components 63. In the retinas of AMD patients, necroptotic retinal cell death was identified by elevated MLKL immunofluorescence 60. Notably, the immunofluorescent intensity of MLKL was well correlated with the amount of lipofuscin accumulation.

Oxidative stress-induced death of RPE cells is recognized as the leading theory of AMD pathogenesis 64. Sodium iodate is extensively used to induce oxidative stress in pre-clinical animal models of AMD 49. Emerging evidence demonstrates that necroptosis is widely involved in sodium iodate-induced RPE and photoreceptor cell death (Table 1) 49, 64, 65. Following sodium iodate injection, RIPK1 catalytic activity increases in rat retinas, with the thickness of the outer nuclear layer (ONL) being reduced in a time-dependent manner 65. RIPK3 aggregation is also detected in RPE cells 49. Moreover, the release of HMGB1 was observed post sodium iodate administration 66. HMGB1 translocation is thought to activate both innate and adaptive immunity, further stimulating necroptosis and many of the harmful immunologic responses thereto 67. Interestingly, Ding *et al.* reported that deletion of thyroid hormone receptors (THRs) significantly suppressed the sodium iodate-induced upregulation of RIPK1/3 and MLKL and, concomitantly, diminished RPE and photoreceptor cell loss 66. This finding indicates a role of THR signaling in necroptosis and the potential of suppressing THR signaling for treating dry AMD.

In response to oxidative stress induced by H_2_O_2_ or tert-butyl hydroperoxide (tBHP), necroptosis regulated by RIPK1/3 was found to be a major type of cell death in RPE cells 58. Hypoxia insult also induced necroptosis of microglia, inducing explosive release of fibroblast growth factor 2, and causing retinal angiogenesis, which is the main pathological change in wet AMD 29. The selective deletion of *Ripk3* in microglia could significantly reduce retinal neovascularization, suggesting it as a promising anti-angiogenic target for treating retinal neovascular diseases 29.

Necroptosis was also observed in dsRNA (a component of drusen in AMD)-induced RPE degeneration 53. In this study, knockout of RIPK3 was able to prevent both cell loss and inflammation. A recent study indicated a lipofuscin-triggered necroptosis in AMD retina 60. Further pharmacological experiments revealed that the necroptotic pathway activated by lipofuscin is distinct from canonical RIPK-dependent signaling and can be blocked with a chemical inhibitor of MLKL instead of RIPK1/3 60. Further clarification of the necroptotic pathways and their roles in AMD based on clinico-pathological analysis can aid discovery of new therapeutic target for retinal degenerative diseases.

### Glaucoma

Glaucoma is also a leading cause of irreversible visual loss and blindness 68. Loss of RGCs induced by I/R injury following intraocular pressure (IOP) elevation and reduction is one of the crucial elements in the pathophysiology of glaucoma 28. The role of necroptosis as a pathogenic factor in glaucoma is documented by multiple studies demonstrating that I/R injury triggers activation of the RIPK signaling pathway, which induces the loss of RGCs 28, 69-71. In retinal tissues from glaucoma patients, typical necroptotic morphological abnormalities (cell swelling and membrane rupture) and significantly elevated expressions of RIPK1 have been revealed by TEM and immunohistochemistry in RGCs 56. It was reported that necroptotic signaling, including RIPK1/3 and MLKL, was significantly activated in an *in vitro* model of retinal I/R injury 72-74. *In vivo* studies have also supported that both RIPK1 and RIPK3 are highly expressed in the ganglion cell layer (GCL) of I/R-injured retinas 69, 75. Interestingly, Jeon *et al*. demonstrated that systemic hypotension with normal IOP could trigger angiotensin II-associated glial cell activation that subsequently caused RGC necroptosis, suggesting a new underlying cause of RGC loss in glaucoma 76.

RSK3 (ribosome S6 kinase 3, a Ser/Thr kinase) reportedly plays a crucial role in the phosphorylation of RIPK3 in retinal necroptosis after retinal I/R injury 28. In this report, RSK inhibition with small interfering RNA or chemical inhibitor downregulated the phosphorylation of RIPK3 and reduced necroptosis both *in vitro* and *in vivo*. The interaction between extracellular signal-regulated kinase (ERK) and RIPK3 is also associated with I/R-induced retinal necroptosis 77. Downregulation of ERK phosphorylation was shown to decrease RIPK3 accumulation, whereas RIPK1 accumulation was not affected, indicating a RIPK1-independent pathway 77. Upon retinal ischemic injury, RIPK3 can phosphorylate DAXX at Ser-668 to induce its translocation from nucleus to cytoplasm, leading to cell death with plasma membrane rupture 78. In hippocampal neurons, the inhibition of RIPK1 blocked this RIPK3-DAXX interaction and subsequent DAXX translocation to cytoplasm 79. However, the mechanism by which RIPK1 regulates the interaction between RIPK3 and DAXX upon retinal I/R injury is still unknown.

Calpains are a group of calcium-dependent proteases that are activated by increased cytosolic calcium during cell death 80. Our own previous studies indicated that *in vitro* retinal I/R injury induced calpain-regulated necrosis of RGCs 81-83. Recent research has revealed that the activity of calpain is regulated by peptidyl-prolyl isomerase 1 (PIN1) and calcium/calmodulin-dependent protein kinase II (CaMKII) in the presence of excessive glutamate, which has been proposed to induce excitotoxicity to mediate the loss of RGCs 9, 80, 84. Retinal I/R injury can trigger a newly identified form of cell death that integrates elements of necroptosis, pyroptosis, and apoptosis 59, 85. This new combined form of cell death, termed PANoptosis, has been deeply investigated in infectious diseases and is believed to be regulated by upstream master molecules Z-DNA binding protein 1 (ZBP1) and transforming growth factor beta-activated kinase 1 (TAK1) 86. There is little evidence yet to identify the upstream regulators of PANoptosis during retinal I/R injury, even in whole neural injury. In the future, it is worth identifying the master regulator of PANoptosis to develop inhibitors that target multiple cell death pathways in the retina and wider nervous system.

Glaucoma is also characterized by optic nerve (ON) degeneration, which is thought to precede the death of RGCs or any clinical manifestations 87. It was found that expression of RIPK1/3 was significantly elevated following ON crush models 27. Of note, caspase inhibition alone failed to provide protection and, in fact, unexpectedly exacerbated RGC necrosis of in ON injured retinas. In contrast, inhibition of RIPKs in combination with caspase blockade delayed both apoptosis and necroptosis of RGCs 27. Moreover, inhibition of RIPK1 promoted moderate axon regeneration that was only minimally affected by caspase blockade 27. These various reports, taken together, indicate RIPK-regulated necroptosis as being an important driver of glaucoma's pathophysiology.

### Retinal detachment

Retinal detachment (RD) is defined as the physical separation of the neurosensory retina from the underlying RPE layer 24, 88. Although the retina can be surgically reattached, the visual acuity of the RD patients is not always restored due to continuous cell death of photoreceptors 89. It was indicated that the activation of caspases and apoptosis were increased following RD 90. However, pan-caspase inhibition fails to prevent the RD-induced photoreceptor loss 91. Further research revealed that necroptotic signaling was activated after RD 14, 92. Intriguingly, pan-caspase inhibition even shifted RD-induced photoreceptor death from apoptosis to necroptosis 14. In contrast, inhibition of RIPK1 in combination with caspase blockade significantly prevented RD-induced photoreceptor death and the reduction of retinal thickness and function 14, 54. *Ripk3* deficiency also exhibited a protective effect on photoreceptors following RD 14. The inhibition of RIPK1 was also able to reduce autophagic cell death in RD when caspases were inhibited 93. Intriguingly, Ding *et al*. reported that induction of autophagy in RD reduced necroptosis of photoreceptors and protected the retina 55. The crosstalk between autophagy and necroptosis was also observed in ischemic stroke and cancer; however, information about this issue in retinal degenerative diseases is still sparse 94-96.

### Retinitis pigmentosa

Retinitis pigmentosa (RP) represents one of the most studied inherited retinal degenerative diseases 97. In RP, vision loss typically begins with night blindness due to rod dysfunction and loss, then further deteriorates due to cone cell death 98-100. In genetic mutation models of RP, cone photoreceptor loss is widely characterized to be driven by necroptosis, whereas rod photoreceptor loss has been shown to occur through apoptosis 101, 102. Adaptive optics scanning laser ophthalmoscopy of RP patients' eyes indicated significantly increased necrotic enlargement of cone photoreceptor cells with concomitant release of HMGB1 within the vitreous humor 100.

Inhibition of RIPK1/3 achieved significant preservation of cone cells in the mouse model of RP (*rd10* mice) 103. Sato *et al*. reported that necroptosis contributed to both cone and rod photoreceptor degeneration in RP mice lacking interphotoreceptor retinoid-binding protein (IRBP) 104. This was confirmed in RP patients with early and progressive photoreceptor abnormalities 104. Another study revealed a bystander effect of rod photoreceptor necroptosis based on a P23H rhodopsin mutant rat, a model representing autosomal dominant RP 105. The study identified that rod photoreceptors principally died through necroptosis, with subsequent release of bioactive molecules (ATP and HMGB1), which may lead to NLRP3 inflammasome activation and drive further bystander cell death of cone photoreceptors.

Microglia also experience necroptosis in the rd1 mouse model of RP 106. This necroptotic process was associated with the activation of Toll-like receptor-4 (TLR4), which recruited Toll/IL-1R domain-containing adaptor-inducing IFNβ (TRIF) *via* its homology interaction motif (RHIM) domain, thereby promoting its interaction with RIPKs 106, 107. Based on these studies, inhibition of necroptosis may be employed as a potential strategy to prevent or delay retinal degeneration in RP.

### Retinal light damage

While the normal daily level of light (380-780 nm; 400-700 nm on primate' retinas) acts on the retina to induce vision and modulate circadian rhythms, excessive light has been reported to cause retinal cell death 108-111. It was shown that light impinging on the retina could cause retinal cell death by interacting with mitochondrial constituents to generate reactive oxygen species (ROS) and trigger apoptosis 112, 113. Mitochondrially-associated necroptosis of RGCs was also identified in blue light-induced retinal damage 51. This necroptosis was able to be attenuated by the siRNA-mediated knockdown of *Ripk1*. Another study demonstrated that blue light could induce an AIF-mediated retinal necroptosis, which was regulated by the phosphorylation of CaMKII-Drp1 cascade 30, 114.

A study compared the retinal cell deaths induced by light and sodium azide, which is an inhibitor of cytochrome oxidase functioning in the mitochondrial electron transport chain 115. The authors found that sodium azide killed RGCs mainly through apoptosis, whereas light-induced cell death occurs mainly *via* necroptosis and could be inhibited by RIPK1 blockade. More powerful light stress (5500 lux, fluorescent white light for 2 to 4 hours) activated Fas ligand (FasL)-Fas signaling and induced apoptosis of photoreceptor cells *via* a paracrine mechanism 116. However, blocking FasL or caspase-8 failed to improve overall photoreceptor survival because of a subsequent hyper-phosphorylation of RIPK1 and compensatory activation of necroptosis 116. Hence, efforts to prevent RGC and photoreceptor loss from light stress should pay attention to necroptosis signaling.

### Ocular blast injury

Ocular blast injury is a major medical concern for both military and civilian victims of explosions due to poor visual outcomes 117, 118. According to the Birmingham eye trauma terminology system (BETTS), ocular blast injuries are mainly classified into two groups - closed-globe injuries and open-globe injuries 119, 120. Closed-globe injury induces retinal cell loss to all layers of the retina followed by nonapoptotic cell death 121. Increased labeling for RIPK1/3 and MLKL was observed in the retina following blast injury, specifically at the injury site, suggesting that necroptosis contributes to an ongoing neurodegenerative response post blast 121-123. However, a study based on repeated primary ocular blast injury in mice indicated that intravitreal injection of RIPK1 inhibitor or vehicle both increased vitreous inflammation and aggravated the RGC degeneration at 28 days post injury 124. The combination of blast injury and intravitreal injection might exacerbate the RGC damage, suggesting intravitreal injection was not an ideal method for anti-necroptotic drug delivery after closed-globe injury.

Open-globe injury refers to perforation or penetration injuries to the eye 125, 126. Severe posterior injuries occur after open-globe injury, including occasional RDs, large RPE vacuoles, activation of microglia, and severe regional photoreceptor cell death 125, 127-129. Significant elevated labeling for markers of necroptosis (RIPK1 increased in the ONL, INL, and Müller glia; RIPK3 increased in the ONL, INL, IPL, and GCL), but not apoptosis, was observed in the retina following open-globe injury, indicating that necroptosis was widely activated in the retina after this injury 125. In addition, open-globe injury can also cause traumatic glaucoma. Thus, early intervention against retinal necroptosis is needed to prevent exacerbation of retinal damage following open-globe injury 130.

### Cytomegalovirus retinitis

Cytomegalovirus (CMV; a ubiquitous DNA herpes virus) retinitis is a potentially blinding manifestation in patients with CMV infection and immune dysfunction 131, 132. Using a mouse model of murine CMV (MCMV) retinitis with retrovirus-induced immunosuppression, Chien and Dix demonstrated that the expression of RIPK1/3 significantly increased in MCMV-infected eyes 133. Notably, increased RIPK3 expression was observed in RPE cells, microglia/macrophages, and glial cells, but not in RGCs in MCMV-infected eyes 134. Depletion of RIPK3 not only inhibited cell death *via* necroptosis during MCMV retinitis, but also significantly attenuated both caspase 3-dependent and AIF-mediated, caspase 3-independent apoptosis 134, 135. In addition, a study using *Bax*^-/-^ mice indicated that *Bax* gene deletion caused more caspase 3-independent cell death of uninfected bystander retinal cells and more cleaved RIPK1, suggesting that BAX might have an important role in the prevention of necroptosis 136. More studies using chemical inhibitors or genetic ablation of apoptotic and necroptotic molecules will help to characterize the mechanism of interaction between apoptosis and necroptosis during CMV retinitis.

### Others

Necroptosis is also linked to cone photoreceptor degeneration in Leber congenital amaurosis (LCA) 11, 137. Analysis of cell death pathways revealed that expressions of necroptotic factors (*Ripk3*, *Tnf-α, Tnfra1*, *Mlkl*, and *Tradd*) were increased in the retinas of LCA mice (*Rpe65*-deficient) 137. Of note, deficiency of type 2 iodothyronine deiodinase (Dio2) provided a neuroprotective effect against necroptosis in retinas of LCA mice 137.

Achromatopsia is an inherited retinal disease characterized by early loss of cone photoreceptors and later rod photoreceptor loss 31, 138, 139. In a zebrafish model of human achromatopsia (*pde6c^w59^*), cone photoreceptors expressed high levels of RIPK1/3, whereas rod photoreceptors were immunopositive for caspase-3, indicating activation of apoptosis 31. Furthermore, morpholino-mediated gene knockdown of *ripk3* rescued cone photoreceptor cell death and restored visual function, suggesting that targeting the necroptotic signaling pathway could be an effective therapeutic intervention for achromatopsia 31.

BMI1 (B lymphoma Moloney murine leukemia virus insertion region 1) is a component of the polycomb repressive complex 1, which is required for the proliferation of peripheral retinal progenitor cells and radial growth of the retina 140, 141. Using *Bmi1*^-/-^ mice, Barabino *et al*. demonstrated that loss of *Bmi1* caused rapid necroptotic degeneration of cone bipolar neurons and cone photoreceptors, but not in rods, during post-natal eye development 8. However, the question of whether necroptosis is directly or indirectly repressed by BMI1 has yet to be answered.

DNA-alkylating agents are commonly used as cancer chemotherapeutics, but they potentially trigger retinal degeneration 142, 143. It is reported that necroptosis plays an important role in alkylation-induced photoreceptor cell loss in male mice 142. Moreover, *Ripk3* deletion partially protected photoreceptor cells from alkylation-induced cell death, attenuating necrosis and reducing inflammation 142. Intriguingly, it was shown that *Parp1* knockout conveyed full protection against alkylation-induced photoreceptor degeneration, whereas RIPK3 knockout only had a partially protective effect, suggesting that activation of necroptosis might be secondary to PARP1 activation in alkylation-induced photoreceptor degeneration 142, 144. The crosstalk between RIPK3 signaling and PARP1 activation remains to be elucidated.

Although necroptosis is now established as an important type of RCD in retinal degenerative diseases, many questions are still to be addressed. Herpes simplex virus (HSV)-1 induces acute retinal necrosis and significant elevation in TNF-α expression 145. Similarly, elevated expressions of TNF-α and TNF receptors were closely associated with the severity of diabetic retinopathy 146, 147. Inhibiting TNF-α afforded a protective effect against HSV-1-induced retinitis; however, no one has yet reported an increase in RIPK1/3 or MLKL in these retinopathies, nor has the involvement of necroptosis therein been definitely documented 148. Moreover, glutamate neurotoxicity has been reported to be crucial to retinal cell death in experimental diabetic retina 149. Thus, it is worth confirming glutamate-related necroptosis and identifying the role of CaMKII in the diabetic retina for further treatment of diabetic retinopathy.

## Therapeutic options to target necroptosis in retina

In this section, we highlight the recently discovered compounds (selective inhibitors, drugs, and chemical agents) regulating the necroptotic cascade pathway in the treatment of diverse retinal disorders.

### Compounds targeting RIPK1

**Figure 3:**
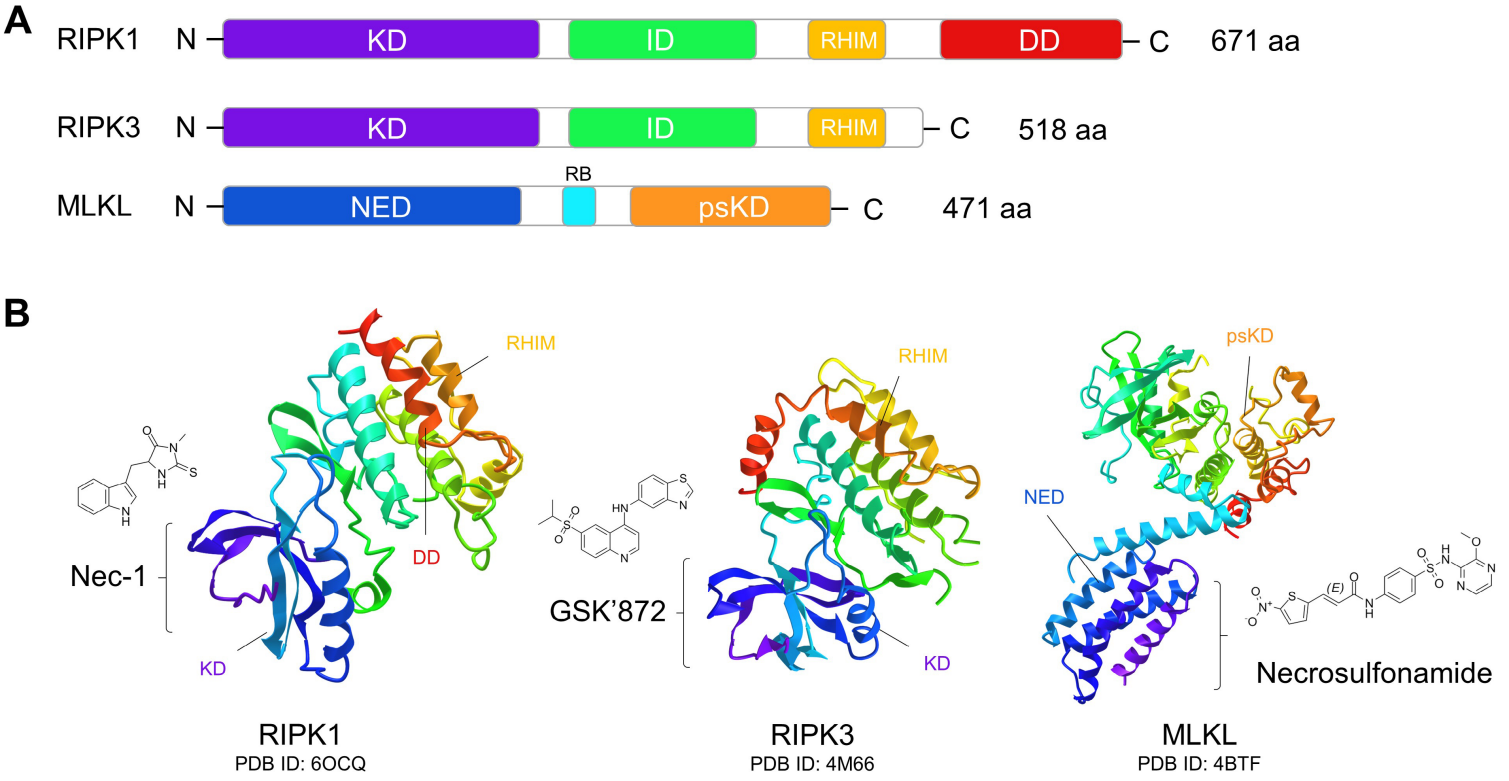
** Domain organization of necroptotic signaling proteins and their targeted chemical inhibitors. (A)** Functional domains of RIPK1, RIPK3, and MLKL. RIPK1 and RIP3 have very similar domain features, both of them containing the N-terminal serine/threonine kinase domain (KD), intermediate domain (ID), and RIP homotypic interaction motif (RHIM) domain. RHIM mediates the assembly of the RIPK1:RIPK3 complex and is crucial for KD activation and induction of necroptosis. RIPK1 also has a C-terminal death domain (DD), which can bind to the death receptors of TNFR1 and Fas to activate apoptosis. In MLKL, the regulatory brace (RB) domain connects the N-terminal execution domain (NED) and C-terminal pseudokinase domain (psKD). The psKD is usually catalytically inactive and contains an unusual pseudoactive site. The NED is sufficient to form channels that can induce membrane depolarization and cell death. **(B)** Tertiary structures of RIPK1, RIPK3, and MLKL and their inhibitors. Nec-1 can bind to the kinase domain of RIPK1 to stabilize it in an inactive conformation. GSK'872 can be caged in the kinase domain of RIPK3 to inhibit its kinase activity. Necrosulfonamide can bind to the N-terminal execution domain of MLKL to block its activation.

Nec-1 is a selective allosteric inhibitor of RIPK1 that binds to its kinase domain and stabilizes it in an inactive conformation (Fig. 3) 102, 150, 151. The protective effect of Nec-1 has been widely proven in pre-clinical models of retinal injuries (Table 2). In an AMD mouse model, Nec-1 treatment significantly counteracted necroptosis of RPE and microglial cells, and inhibited retinal neovascularization 29, 49. Inhibition of pathological angiogenesis by Nec-1 treatment has also been reported in a laser-induced choroidal neovascularization (CNV) model, which was associated with caspase activation and suppression of M2 macrophage polarization 152. Moreover, necrotic RGC death was attenuated by Nec-1 in rodent models of oxygen and glucose deprivation, optic nerve damage, and high IOP, indicating multiple potential protection effects of Nec-1 on glaucoma 69, 71, 153. In addition, Nec-1 has been shown to inhibit necroptosis of multiple retinal cell types across diverse models of retinal detachment, retinitis pigmentosa, and retinal light damage (Table 2). Of particular note, treatment with Nec-1 and a pan-caspase inhibitor conferred near-complete protection of RPE cells against cell death induced by tamoxifen, which is widely used in low dosages as an adjuvant therapy for breast cancer 154.

Nec-1 stable (Nec-1s), a modified derivative of Nec-1, has a higher affinity and specificity for RIPK1 155, 156. In rodent models of retinitis pigmentosa, treatment with Nec-1s significantly protected cone and rod photoreceptors from degeneration and reduced cell loss in ONL 104, 105. Nec-1s also exhibited neuroprotective effects in ocular blast injury 123, 124. Additionally, Nec-1s delayed cone cell death and preserved the remainder of the outer retina in a zebrafish model of achromatopsia 31. However, the clinical application of Nec-1 and Nec-1s is limited due to their moderate potency and poor *in vivo* pharmacokinetic properties 157. The development of compounds that overcome these limitations would be of significant benefit.

The RIPK1-inhibitory compound (RIC) is another inhibitor of RIPK1 that has a distinct chemical profile compared to Nec-1 70, 150. It was reported that RIC effectively suppressed necroptosis of RGC and RPE and had a novel neuroprotective effect in both glaucoma and dry AMD 65, 70. Additionally, the excellent corneal permeability of RIC offers an appealing advantage for its administration by eye drop, making it a good candidate for clinical therapy 65. Several other inhibitors of RIPK1, such as GSK'963, RIPA-56, GSK2982772, and DNL747, have been identified in the past decade 61, 158-161. However, their roles in retinal degenerative diseases are still unknown. Of note, GSK2982772 has been advanced into phase II clinical trials for the treatment of rheumatoid arthritis, plaque psoriasis, and active ulcerative colitis, showing good oral pharmacokinetics and tolerance 162.

### Compounds targeting RIPK3

GSK'872, a specific small molecule inhibitor of RIPK3 binding to its kinase domain, plays an important role in suppressing necroptosis and RIPK3-dependent inflammation 163, 164. It was reported that inhibition of RIPK3 by GSK'872 treatment significantly reduced phosphorylation of MLKL and cytosolic Ca^2+^ concentrations in retinal cells *in vitro* following elevated hydrostatic pressure 72, 165. In addition, the anti-necroptotic effect of GSK'872 has been indicated in osteoclastogenesis and non-alcoholic fatty liver disease *in vitro* and *in vivo* 166, 167. However, the *in vivo* retinal neuroprotective efficiency of GSK'872 still needs to be confirmed.

HS-1371 and HG-9-91-01 are potent inhibitors of RIPK3 and exhibit significant anti-necroptotic effect against TNF signaling 168, 169. However, HG-9-91-01 also triggers RIPK1/3 and caspase 1/8-mediated apoptosis and pyroptosis 169. Recently, Xu *et al.* discovered a new RIPK3 inhibitor, AZD5423, that effectively protected against cisplatin- and I/R-induced acute kidney injury in mice 170. Zharp-99 is another recently discovered RIPK3 inhibitor that efficiently blocks TNF-induced necroptosis in both human and mouse cells 171. Zharp-99 shows promising therapeutic potential, with good *in vitro* safety profiles and *in vivo* pharmacokinetic parameters 171. Further investigations in pre-clinical models of retinal injury using HS-1371, AZD5423, and Zharp-99 will provide crucial insights for developing novel therapies for necroptotic retinopathy.

### Compounds targeting MLKL

Necrosulfonamide (NSA) is a selective inhibitor of MLKL that blocks its N-terminal CC domain function 35. NSA suppresses necroptosis by directly inhibiting necrosome formation, showing protective effects against necroptosis in pulmonary I/R injury, spinal cord injury, and Alzheimer's disease 172-176. In retinal degenerative disease, NSA showed an ameliorative effect in a pre-clinical animal model of Achromatopsia through alleviating necroptotic degeneration of cone photoreceptor 31. However, the therapeutic potential of NSA in other retinal disorders is yet to be demonstrated. GW806742X is a potent MLKL inhibitor that binds the pseudokinase domain of MLKL 177. Treatment with GW806742X and other RIPK1/3 inhibitors significantly blocked necroptosis and inflammation 178, 179. BI-8925, another potent MLKL inhibitor identified in TNFα-induced necroptosis, works by stabilizing the inactive state of MLKL by an essential π-π stacking interaction 180. Since GW806742X and BI-8925 are novel inhibitors of MLKL, their potential effects on retinal degenerative diseases need to be addressed in the future.

### Others

Ma *et al.* reported that anti-thyroid drugs (1% sodium perchlorate monohydrate and 0.05% methomazole in drinking water) reduced retinal necroptosis and oxidative stress responses in a mouse model of AMD and LCA 64, 137. High serum TH levels have been correlated with increased risk of AMD, supporting the clinical therapeutic potential of anti-thyroid treatment 64.

Rapamycin, the first generation of mTOR inhibitors, can also inhibit the necroptosis of photoreceptors in retinal detachment by decreasing the RIPK1 expression and AIF nuclear translocation 55. However, its clinical application is limited due to poor water solubility and stability. Fortunately, rapamycin has a number of analogs (rapalogs) with improved pharmacokinetic properties 181. These may be explored in retinal degenerative diseases in the future.

Melatonin is an important hormone that has a wide-ranging neuroprotective effect and can enhance cognitive function in clinical trials 182, 183. In a model of acute glaucoma, melatonin treatment attenuated necroptosis of RGCs, reduced retinal thinning, and ameliorated retinal dysfunction 184. Interestingly, apoptosis and pyroptosis have also been inhibited by melatonin, suggesting a multi-protective potential for therapeutic application 184. Minocycline, another promising clinical neuroprotective agent for acute stroke patients, also exhibited an anti-necroptotic effect on RPE cell loss caused by blue light 185, 186. Although both of melatonin and minocycline are clinically used and have antioxidant and anti-inflammatory effects, their targeting mechanism in the necroptotic signaling pathway requires further exploration 187, 188.

As mentioned above, compounds that inhibit key effector molecules within the necroptotic signaling pathway also show protective effects on retinal cells, such as KN-93 (CaMKII), Mdivi-1 (DRP1), and LJH685 (RSK) (Table 2) 28, 30. However, the efficacy and safety of these compounds still need to be evaluated in *in vivo* pre-clinical models of retinal degenerative diseases.

The human bone marrow-derived mesenchymal stem cell (bmMSC) secretome also exhibits a versatile neuroprotective ability 189. Among its documented effects are: inhibition of RIPK1/3, MLKL, and apoptotic effectors; modulation of autophagy; and activation of antioxidant machinery, all of which were observed in a model of spontaneous retinal neurodegeneration 190. Although the neuroprotective effect of MSC-based therapy has been approved for the treatment of retinal neurodegeneration, the specific anti-necroptotic compounds within the bmMSC secretome remain unidentified 191, 192.

Of these compounds that can regulate necroptosis in models of retinal degenerative diseases, some are administered prophylactically (as pre-treatment), while others are used therapeutically (as post-treatment) (Table 2). Since necroptosis, as a process, occurs rapidly once triggered, prophylactic treatments with Nec-1 and GSK'872 are effective approaches to inhibit key kinases near the start of the necroptotic signaling cascade 28, 58. Excessive oxidative stress and inflammation are triggers for AMD and retinal light damage 193, 194. Some clinically validated antioxidant and anti-inflammatory compounds, such as anti-thyroid drugs and minocycline, may also mitigate retinal cell death by preventing the triggers, although their specific targets in necroptotic signaling, if any, remain to be elucidated 64, 184.

An effective post-treatment often indicates therapeutic potential of the compound 70, 187. Because necroptosis can release inflammatory effectors, bystander inflammation may occur for a long time following cell death and lead to subsequent damage and necroptosis of adjacent cells 105. Therefore, early intervention with anti-necroptosis compounds after the onset of disease is important to alleviate further retinal tissue damage and dysfunction.

## Conclusion and prospects

The discovery and characterization of necroptosis represents a major breakthrough in the field of cell death and signaling in the past decade. Extensive and increasing evidence demonstrates that numerous retinal degenerative diseases involve necroptosis mediated by RIPK1/3 and MLKL, occurring in different retinal layers and cell types. Moreover, advances in characterizing mitochondrial dysfunctions and mutations in key regulators - such as CaMKII, DRP1, AIF, and PARP1 - in retinal degenerative diseases involving necroptotic signaling have expanded the understanding of organelle dysfunction and the molecular mechanisms of necroptosis.

Inhibition of these master necroptotic regulators by diverse compounds or genetic interventions could potentially attenuate necroptosis of retinal cells. These strategies have already demonstrated multiple benefits in treating retinal degenerative diseases, such as reducing ocular inflammation, inhibiting retinal neovascularization, and preventing optic nerve damage. Notably, several compounds approved in clinical trials, such as GSK2982772, methomazole, rapalogs, melatonin, and minocycline also exhibited anti-necroptotic abilities in retinal degenerative diseases.

Although we have learned much about necroptosis, its role in necrotizing herpetic and diabetic retinopathy remains enigmatic. Current knowledge about necroptosis in retinal degenerative diseases mainly comes from animal models, with clinical trials of these anti-necroptosis strategies in patients still in their early days. Development of safe necroptosis inhibitors, stem cell-based therapy, and genetic strategies for targeting master regulators of necroptosis remain the key points of future clinical development.

## Figures and Tables

**Table 1:** Necroptosis in models of retinal degenerative diseases and injuries

Retinal disorders	Disease models	Cell types	Related molecules	Descriptions	Reference
AMD	Sodium iodate insult	RPE	RIPK1, RIPK3, MLKL, HMGB1	Sodium iodate insult induced necroptosis of RPE cells in the damaged and undamaged junction areas in retina, which displayed folding of the outer nuclear layer and shortened outer segment areas.	49, 64, 66
	H_2_O_2_/tBHP insult	RPE	RIPK3, HMGB1	Oxidative stress induced RIPK3-mediated necrosis as a predominant form of RPE cell death, which showed intracellular PI staining and membrane blebbing.	58
	Hypoxia insult	Microglia	RIPK1, RIPK3, MLKL	Hypoxia activated the RIP1-RIP3-MLKL signaling axis to induce necroptosis of retinal microglia.	29
	dsRNA induction	Photoreceptors, RPE	RIPK3, HMGB1	After dsRNA injection, dying photoreceptors and RPE cells exhibited necroptotic morphology accompanied by swollen vacuoles in the inner segments of the retina.	53
Glaucoma	High intraocular pressure	RGC	RIPK1, RIPK3, ERK1/2, Arginase 1	High intraocular pressure induced necroptosis of RGCs and decreased retinal cell numbers in the GCL and INL/ONL.	69, 70, 195
	Systemic hypotension	RGC	RIPK1, RIPK3, Angiotensin II	Systemic hypotension with a normal IOP triggered necroptosis of RGC, which was associated with angiotensin II-related glial cell activation.	76
	Optic nerve crush	RGC	RIPK1, RIPK3	Optic nerve crush injury caused necroptosis of RGCs, accompanied by elevated expression of RIPK3.	27
	Ischemia and ischemia reperfusion insult	RGC	RIPK1, RIPK3, MLKL, RSK3, Calpain, tAIF, Daxx	Ischemia and ischemia reperfusion insult increased formation of RIPK1-RIPK3 complexes and mitochondrial polarization in RGCs to induce necroptotic death.	28, 78, 82
Retinal detachment	Subretinal injection	Photoreceptors	RIPK1, RIPK3	Retinal detachment caused necroptosis of photoreceptors accompanied by increased expression of RIPK1 and RIPK3.	14, 55, 93
Retinitis pigmentosa	*rd10* mutation	Cone photoreceptor	RIPK1, RIPK3	The retinas of *rd10* mice exhibited a typical necrotic morphology and elevated expression of RIPK3 in cone, but not rod photoreceptors.	103
	P23H rhodopsin mutation	Rod photoreceptor	RIPK1, RIPK3, DRP1	The P23H rhodopsin mutant rat displayed marked upregulation of the RIP1/RIP3/DRP1 axis in rod photoreceptors; cell loss could be rescued by necrostatin-1 treatment.	105
	*Irbp* mutation	Photoreceptors	RIPK1, RIPK3	In the retinas of *Irbp*^-/-^ mouse, RIPK1 and RIPK3-mediated and TNF-induced necrosis contributed to both cone and rod photoreceptor degeneration.	104
	retinal degenerative rd1 mice	Microglia	RIPK1, RIPK3, TLR4	Microglia experienced RIPK1 and RIPK3-dependent necroptosis in the retinal degenerative rd1 mice; *Tlr4* deficiency was able to ameliorate microglial necroptosis.	106
Retinal light damage	Blue light exposure	RGC	RIPK1, RIPK3, AIF	Blue light insult caused a decrease in the viability of RGC-5 cells, with upregulated expressions of both RIPK1, RIPK3, and AIF.	51
	Blue light exposure	Retinal cell	CaMKII, Drp1, AIF	Blue light insult induced AIF-mediated necroptosis of R28 cells with activation of CaMK II-induced Drp1 phosphorylation.	30
	White light insult	Photoreceptors	RIP1	White light insult (5500 lux, 2-4 hours) induced both apoptosis and necrosis in 661W cells; RIPK1 was hyper-phosphorylated when caspase-8 was inhibited.	116
	White light insult	RGC	RIP1	White light insult (1000 lux, 48 hours) induced cell death of RGC-5 cells, which could be attenuated by RIPK1 inhibition.	115
Ocular blast injury	Overpressure airwave blast	Retinal cell	RIPK1, RIPK3	Overpressure airwave blast caused retinal cell death in multiple layers of the retina, with increased labeling for RIPK1 and RIPK3.	121
	Repeated primary ocular blast	RGC	RIPK1, RIPK3	In a repeated primary ocular blast injury mouse model, the RIPK-mediated necroptosis occurred in the GCL, OPL, and IPL of the retina.	124
	Mild blunt trauma	Retinal cell	RIPK1, RIPK3	Blast-induced mild blunt trauma caused necroptotic cell death mediated by RIPK1 and RIPK3 in multiple layers of the retina.	122
	Plastic pellet impact	RGC	RIPK1, MLKL	Following blunt ocular injury caused by plastic pellet impact, MLKL protein expression was upregulated at 48 h in RGCs, inner nuclear cells, and ONL cells.	123
	Blast-induced open ocular trauma	Retinal cell	RIPK1, RIPK3	Blast wave exposure caused necroptotic cell death in multiple layers of the retina; RIPK1 increased in the ONL, INL, and Müller glia, while RIPK3 increased in the ONL, INL, IPL, and GCL.	125
Cytomegalovirus retinitis	Murine cytomegalovirus infection	Retinal cell	RIPK1, RIPK3	In murine cytomegalovirus-infected eyes, necroptotic cell death occurred in multiple layers of retina with increased expressions of TNFα, RIP1K, and RIPK3, whereas *Bax* depletion increased the cleavage of RIPK1.	133, 136
Leber congenital amaurosis	*Rpe65*-deficiency	Cone photoreceptor	RIPK1, RIPK3, MLKL, Dio2, RPE65	In retinas of Leber congenital amaurosis model mice (*Rpe65*-deficiency), cone cells showed severe degenerations associated with necroptosis signaling; *Di*o*2* deficiency reduced necroptosis activity.	137
Achromatopsia	*pde6c^w59^* mutant	Cone photoreceptor	RIPK1, RIPK3, pde6c	In *pde6c^w59^* mutant zebrafish, severe degeneration of cone photoreceptors occurred in the retina, with high expressional levels of RIPK1 and RIPK3.	31
DNA alkylation agent-induced retinal degeneration	Methyl methanesulfonate insult	Photoreceptors	RIPK1, RIPK3	Alkylation agent-induced dying photoreceptors exhibited necrotic morphology and overexpression of RIPK1 and RIPK3.	142

AMD, age-related macular degeneration; RPE, retinal pigment epithelium; RIPK, receptor-interacting protein kinase; MLKL, mixed lineage kinase domain-like protein; HMGB1, molecule high mobility group box-1; tBHP, tert-butyl hydroperoxide; PI, propidium iodide; FGF2, fibroblast growth factor 2; IL, interleukin; RGC, retinal ganglion cell; ERK, extracellular signal-regulated protein kinase; RSK3, ribosome S6 kinase 3; tAIF, truncated apoptosis-inducing factor; DRP1, dynamin-related protein 1; IRBP, interphotoreceptor retinoid-binding protein; TNF, tumor necrosis factor-alpha; TLR4, Toll-like receptor 4; CaMKII, calcium/calmodulin-dependent protein kinase II; GCL, ganglion cell layer; OPL, outer plexiform layer; IPL, inner plexiform layer; ONL, outer nuclear layer; INL, inner nuclear layer; Dio2, type 2 iodothyronine deiodinase; PDE6C, phosphordiesterase 6C.

**Table 2:** Anti-necroptosis compounds for treating retinal diseases

Compounds	Targets	Related diseases	Treatment	Affected cell types	Related molecules	Reference
Nec-1	RIPK1	AMD	Pre/post-treatment	RPE, Microglia	RIPK3, MLKL	29, 49
		Glaucoma	Pre/post-treatment	RGC	RIPK3	27, 69, 71, 153
		Retinal detachment	Pre/post-treatment	Photoreceptors	AIF	14, 54
		Retinitis pigmentosa	Post-treatment	Photoreceptors, Microglia	RIPK3, DRP1	103-106
		Retinal light damage	Pre/post-treatment	RGC, Photoreceptors	RIPK3, CaMKII, Drp1, AIF	30, 51, 115, 116
		Tamoxifen toxicity	Post-treatment	RPE	N.P.	154
Nec-1s	RIPK1	Ocular blast injury	Post-treatment	RGC	RIPK3, MLKL	123, 124
		Achromatopsia	Post-treatment	Cone photoreceptor	RIPK3	31
RIC	RIPK1	AMD	Post-treatment	RPE	N.P.	65
		Glaucoma	Post-treatment	RGC	RIPK3	70
GSK′872	RIPK3	Retinal ischemic stress	Pre-treatment	RGC	MLKL	72
Necrosulfonamide	MLKL	Achromatopsia	Post-treatment	Cone photoreceptor	RIPK1, RIPK3	31
ALLN	Calpain	Glaucoma	Pre-treatment	RGC	AIF	81, 82
KN-93	CaMKII	Retinal light damage	Post-treatment	Retinal R28 cells	Drp1, AIF	30
Mdivi-1	Drp1	Retinal light damage	Post-treatment	Retinal R28 cells	CaMKII, AIF	30
LJH685	RSK3	Retinal ischemic stress	Pre-treatment	RGC	RIPK3	28
U0126	ERK	Glaucoma	Post-treatment	RGC	RIPK3	77
Melatonin	N.P.	Glaucoma	Post-treatment	RGC	RIPK1, RIPK3	184
Anti-thyroid drugs	N.P.	AMD	Pre-treatment	Photoreceptors, RPE	RIPK1, RIPK3, MLKL	64
		LCA	Post-treatment	Cone photoreceptor	RIPK1, RIPK3	137
Nec-7	N.P.	AMD	Pre-treatment	RPE	RIPK3	58
Rapamycin	N.P.	Retinal detachment	Post-treatment	Photoreceptors	RIPK1, AIF	55
Trolox	N.P.	Retinal light damage	Post-treatment	Retinal R28 cell	CaMKII, Drp1, AIF	30
Minocycline	N.P.	Retinal light damage	Pre-treatment	RPE	RIPK3	186

Nec, Necrostatin; RIPK, receptor-interacting protein kinase; AMD, age-related macular degeneration; RPE, retinal pigment epithelium; MLKL, mixed lineage kinase domain-like protein; RGC, retinal ganglion cell; AIF, apoptosis-inducing factor; CaMKII, calcium/calmodulin-dependent protein kinase II; DRP1, dynamin-related protein 1; N.P., not provided; Nec-1s, Nec-1 stable; RIC, RIPK1-inhibitory compound; AMD, age-related macular degeneration; ALLN, N-acetyl-leucyl-leucyl-norleucinal; DRP1, dynamin-related protein 1; RSK3, ribosome S6 kinase 3; ERK, extracellular signal-regulated protein kinase; LCA, Leber congenital amaurosis.

## Abbreviations

TNFR1: tumor necrosis factor (TNF)-receptor 1
TRAF2: TNF receptor associated factor 2
cIAP1/2: cellular inhibitor of apoptosis proteins 1 and 2
LUBAC: linear ubiquitin chain assembly complex
TAK1: TGF-activated kinase 1
IKK: IκB kinase
TAB2/3: TAK1-binding protein 2/3
NEMO: NF-κB essential modulator
cFLIP: cellular FLICE-like inhibitory protein
NF-κB: nuclear factor-κB
FADD: Fas-associated protein with death domain
RIPK: receptor-interacting protein (RIP) kinase
complex IIa-RIA: complex IIa-RIPK1-independent apoptosis
complex IIa-RDA: complex IIa-RIPK1-dependent apoptosis
MLKL: mixed-lineage kinase domain-like pseudokinase
DAMP: damage-associated molecular pattern
ESCRT-III: endosomal sorting complexes III
PGAM5: phosphoglycerate mutase family member 5
DRP1: dynamin-related protein 1
ROS: reactive oxygen species
tAIF: truncated apoptosis-inducing factor
